# Born with an advantage: early life and maternal effects on fitness in female ground squirrels

**DOI:** 10.1093/beheco/arae013

**Published:** 2024-02-28

**Authors:** Tanner Yuen, Kathreen E Ruckstuhl, April R Martinig, Peter Neuhaus

**Affiliations:** Department of Biological Sciences, University of Calgary, 2500 University Drive NW, Calgary, Alberta, T2N 1N4Canada; Department of Biological Sciences, University of Calgary, 2500 University Drive NW, Calgary, Alberta, T2N 1N4Canada; Zoology Department, University of Cambridge, Downing Street, Cambridge, CB2 3EJ Cambridgeshire, UK; Department of Biological Sciences, University of Calgary, 2500 University Drive NW, Calgary, Alberta, T2N 1N4Canada; Evolution & Ecology Centre and School of Biological, Earth and Environmental Sciences, 12 UNSW, Sydney, Australia; Department of Biological Sciences, University of Calgary, 2500 University Drive NW, Calgary, Alberta, T2N 1N4Canada; Zoology Department, University of Cambridge, Downing Street, Cambridge, CB2 3EJ Cambridgeshire, UK

**Keywords:** Columbian ground squirrels, lifetime reproductive success, longevity, life-history trade-offs, maternal effects

## Abstract

Lifetime fitness and its determinants are an important topic in the study of behavioral ecology and life-history evolution. Early life conditions comprise some of these determinants, warranting further investigation into their impact. In some mammals, babies born lighter tend to have lower life expectancy than those born heavier, and some of these life-history traits are passed on to offspring, with lighter-born females giving birth to lighter offspring. We investigated how weight at weaning, the relative timing of birth in the season, maternal weight, and maternal age affected the longevity and lifetime reproductive success (LRS) of female Columbian ground squirrels (*Urocitellus columbianus*). We hypothesized that early life conditions such as offspring weight would not only have lifetime fitness consequences but also intergenerational effects. We found that weight at weaning had a significant impact on longevity, with heavier individuals living longer. The relative timing of an individual’s birth did not have a significant association with either longevity or LRS. Individuals born to heavier mothers were found to have significantly higher LRS than those born to lighter mothers. Finally, maternal age was found to be significantly associated with their offspring’s LRS, with older mothers having less successful offspring. Our results provide evidence that early life conditions do have lifelong fitness and sometimes intergenerational consequences for Columbian ground squirrels.

## Teaser

In animals, including humans, it has been shown that aspects of early life conditions may impact an individual’s life in general. We investigated the impact of the body condition of juvenile Columbian ground squirrels, time of reproduction, and the condition and age of the mother on longevity and lifetime reproductive success in female offspring. Our results confirmed significant impacts of age and weight of mothers and juvenile condition at weaning on reproduction and lifespan of female offspring.

## INTRODUCTION

The effects of early life conditions on fitness have been widely studied in many species, ranging from maternal condition to social status significantly affecting the ontogeny and life history of offspring (Lansing 1947; Lummaa 2003; Descamps et al. 2008; Rödel et al. 2009; Magnabosco et al. 2016; Martinig et al. 2020). Life-history traits describe factors that influence the pattern of reproduction and survival in an organism (Stearns 1989).

Life-history traits of an organism early in life can have a major impact on the lifetime fitness of that organism. For example, in humans (*Homo sapiens*), it has been shown that early conditions can have a large impact on lifetime fitness (Lummaa 2003). Low birth weights in newborn humans have been linked with longevity-reducing diseases such as type 2 diabetes and coronary heart disease (Phillips et al. 1994; Eriksson 2001). Adverse conditions early in life have also been noted to reduce metrics of fitness in a range of other species, for example, in European rabbits (*Oryctolagus cuniculus*) where litter size, mother’s age, and offspring date of birth impact fitness (Rödel et al. 2009); in yellow baboons (*Papio cynocephalus*) where cumulative early life effects, such as population density, a mother’s social rank, or competition from siblings affect longevity, with those experiencing at least three adverse conditions living significantly shorter lives (Tung et al. 2016); in yellow ground squirrels (*Spermophilus fulvus*), where weaning weight was the main determinant of survival to reproductive age (Vasilieva and Tchabovsky 2020); or in domestic swine (*Sus scrofa domesticus*), where lighter-born individuals experience reduced productivity and longevity (Magnabosco et al. 2016).

Maternal age is another early life condition that may influence the life history traits of offspring. One proposed trend is the “Lansing effect,” in which decreasing offspring longevity is associated with increasing maternal age, which was first described in rotifers (*Rotifera spp.*) (Lansing 1947). In spotted hyenas (*Crocuta crocuta*), early life effects, such as the age of an individual’s mother, have been shown to influence fitness, with individuals born to prime-age mothers having greater longevity and lifetime reproductive success (LRS) (Gicquel et al. 2022). Similarly, maternal age in European rabbits was reported to affect offspring LRS, with primate-aged females producing offspring with higher LRS, while survival to maturity increased linearly with the mother’s age (Rödel et al. 2009).

The season or timing of an individual’s birth can also affect lifetime fitness. In a 19th-century population of people in Canada, being born in November to March, June, or September was shown to positively influence reproductive success, with mothers born in these months having more children on average than those born in other months of the year (Lummaa and Tremblay 2003). Birth month has also been shown to affect lifespan in humans (Doblhammer and Vaupel 2001), while the underlying proximate cause of birth month remains unclear.

Columbian ground squirrels (*Urocitellus columbianus*) are a well-studied species of herbivorous, hibernating, semi-fossorial colonial rodent, with many aspects of their ecology, physiology, and life history being well documented (Boag and Murie 1981; Dobson 1992; Neuhaus et al. 2004). This wealth of literature, as well as some aspects of their biology, make Columbian ground squirrels an excellent species for the study of life-history evolution and ecology. Columbian ground squirrels are small mammals, facilitating relatively easy capture and handling of the animals during data collection.

The colonial nature of the species allows for the collection of data from a large sample of individuals who share a very similar environment and allows for long-term monitoring over an individual’s lifespan. Columbian ground squirrels are a relatively long-lived rodent, with an average lifespan of 5 years in females, and some living more than ten years (Neuhaus and Pelletier 2001). This species has relatively small litters (mean around three juveniles/year), and a short active season, spanning approximately 4 months of the year while hibernating for the other 8 months. Adults emerge from hibernation in spring, typically in early or mid-April, mating shortly afterwards. Females gestate for approximately 24 days and nurse their young for approximately 27 days (Murie and Harris 1982). After weaning, the young emerge and begin to feed on vegetation. The entire colony is typically back in hibernation by the end of August, ending the active season (Murie and Harris 1982).

In this study, we investigated the effects of multiple early life conditions in female offspring: an individual’s weight at weaning, the relative timing of birth, the weight of the individual’s mother at emergence from hibernation, and the age of the individual’s mother at the time of birth on an individual’s LRS and longevity. We hypothesized that early life conditions will have a significant impact on the lifetime fitness of an individual. Specifically, we predicted that individuals weaned lighter would be expected to live shorter lives and produce fewer offspring than those weaned heavier, as a previous study showed that body weight at birth and at weaning is important in the survival of Columbian ground squirrel neonates, or for individual LRS in yellow ground squirrels (Neuhaus 2000b; Vasilieva and Tchabovsky 2020).

Additionally, we expected that individuals born later in the season would live shorter lives and have fewer offspring than those born earlier in the season because earlier-born individuals would have more time to accumulate resources and grow before hibernation. This prediction is supported by research on European rabbits (*Oryctolagus cuniculus*), which indicates that individuals born later in the birthing season display reduced LRS and have shorter lives (Rödel et al. 2009).

In Columbian ground squirrels, spring body condition is strongly correlated with spring body mass (*r* = 0.83), while structural size (such as zygomatic breadth) is not. In addition, there is a correlation between spring body mass and litter mass (*r* = 0.37) and litter size (*r* = 0.29) (Dobson et al. 1999). We thus expected that female offspring born to heavier mothers will live longer and have more offspring over their lifetime than those born to lighter mothers. Lastly, a study on Belding’s ground squirrels (*Urocitellus beldingi*) has shown a positive effect of age and experience on maternal territorial behaviors, including aggression and territorial defense (Nunes 2014). Therefore, we predicted that individuals born to older, more experienced mothers would have better survival, greater longevity, and LRS.

## METHODS

We conducted the study on a colony of Columbian ground squirrels in Sheep River Provincial Park in Alberta, Canada (110°W, 50°N) on an open meadow at 1530 m of elevation. We have extensively studied the colony since 1984 (Wiggett and Boag 1986), with our study encompassing all of the years between 2007 and 2022. We began observing the meadow each year in early April and ended after juvenile emergence from the natal den in early July. Columbian ground squirrels are hibernating for up to 9 months of the year (Neuhaus 2000a). This time constraint allows them to have only one litter per year (no second or replacement litter). Overwinter mortality is fairly high up to yearling age, after which most deaths occur during the active season (Neuhaus and Pelletier 2001). During the active season, they are exposed to a multitude of avian and terrestrial predators (Betts 1976). In our study area, females emerge around mid to late April (variable due to environmental conditions—Murie and Harris 1982) and come into estrus for one day, on average 4 days after emergence. During estrous females will end up mating in underground burrows with multiple males (Raveh et al. 2010). We confirmed a female’s mating date through observations of mating activity and through visible caking or sperm plugs at capture. After conception, females become territorial; gestation is 24 days after which they give birth to 1–8 naked and blind juveniles (average 2–4) in a specially dug natal burrow (Murie and Harris 1982). Females have five pairs of teats (Moore 1961), and competition for milk exists (Skibiel et al. 2009). Approximately 27 days after parturition, the offspring emerges above ground, are weaned, and become independent. Some females reproduce the first time as yearlings, but most do not reproduce until the age of two (Neuhaus et al. 2004).

We trapped individuals ad libitum using 15 × 15 × 48 cm and 13 × 13 × 40 cm National and Tomahawk traps baited with peanut butter, starting at the time of emergence from hibernation in early April. All individuals in the colony were captured at emergence and weighed to the nearest 5 g using a Pesola © spring scale in the first 2 to 3 days after emergence. We marked all individuals with black hair dye on their backs for identification from a distance, as well as with unique fingerling fish tags applied to each ear to allow permanent identification. Individuals were then captured regularly to note reproductive status and weight. We know the age of most individuals because they had been caught and marked as juveniles within 1 day of emergence from their natal burrow. Maternity was also confirmed by microsatellite analysis of an ear tissue sample taken from each offspring (following the methods described in Raveh et al. (2010)).

In this study, we only assessed the life-history traits of female ground squirrels. Females are philopatric, whereas most males emigrate as yearlings (Wiggett and Boag 1992; Neuhaus 2006). We estimated the longevity of an individual by determining the number of years the individual was present in the dataset, and we considered the disappearance of the individual to signal death, an assumption that is reasonable because females do not disperse. We defined the lifetime reproductive success (LRS) of individuals as the number of offspring that reached yearling age produced over an individual’s lifetime. Because males do hibernate in their natal colony before dispersing, the LRS measure included yearling males. Only adult females who had died were included in these analyses.

We used two generalized linear mixed models to assess the influences of maternal age, maternal weight, offspring weight at weaning, and time of birth on female lifetime fitness: the first model was used to assess the associations with longevity (*n* = 518 individuals), and the second to assess associations with LRS (*n* = 115 individuals).

We chose a generalized linear mixed model due to its ability to assess the influences of multiple variables whilst being robust to overdispersion in the data. We used the “glmmTMB” package, version 1.1.7, in R, version 4.1.2, as it allowed for the specification of a negative binomial distribution to account for non-normal data and overdispersion (Brooks et al. 2017; R Core Team 2021).

We standardized all fixed effects using a z-transformation:


$$\begin{array}{l} \frac{x-mean\left(x\right)}{sd\left(x\right)} \end{array}$$

where *×* is the fixed effect variable, *mean(x)* is the mean of *x*, and *sd(x)* is the standard deviation of *x*.

We included individual offspring weight at weaning, the timing of birth (Julian date), maternal age (in years), and maternal weight (at emergence from hibernation) as fixed effects when modeling the influences upon longevity and LRS. Because individuals that live longer may have higher reproductive success, we included longevity as a fixed effect when modeling the influences upon LRS.

We defined weight at weaning as the first recorded weight of an individual after emergence from their natal burrow in the year of their birth, as young are born underground and do not emerge or feed on vegetation before weaning. Similarly, we used the date of emergence from the natal burrow (recorded as Julian date; measured in days from the beginning of the calendar year) as an indicator for the timing of birth. We defined maternal age as the age in years of an individual’s mother when the individual was born and defined maternal weight as the weight of the individual’s mother at emergence from hibernation in the year of the individual’s birth. We used emergence weights because they were the most widely available weights, ensuring that all individuals were accounted for in the data.

We considered the year of birth and maternal identity as random effects and included them in the model to account for variation in yearly conditions and maternal variability. We calculated the 95% confidence intervals (CI). We tested for collinearity between predictor variables, considering that collinearity above 0.60 can lead to biased estimates (Freckleton 2011). Lifetime reproductive success and lifespan were highly correlated (Pearson’s *r* = 0.61), meaning that individuals who lived longer had higher lifetime reproductive success. We did not include litter size in our analyses because offspring mass was negatively correlated with litter size (Pearson’s *r* = −0.51; Neuhaus 2000b). We only included a quadratic term for maternal age in our models because it was highly correlated with linear maternal age (Pearson’s *r* > 0.90). All other correlations were below ± 0.40. All analyses were done in R version 4.2.0 (R Core Team 2021), and report means ± standard errors (SE), unless stated differently.

## RESULTS

**Figure 1:**
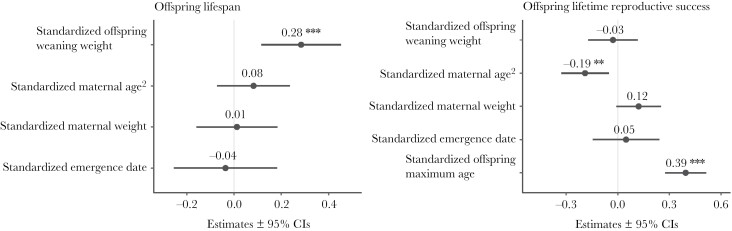
Statistical model outputs (dot-whisker plots), with points representing model regression coefficient estimates and bars representing ± 95% confidence intervals (CIs). Numbers above each dot-whisker indicate the Beta coefficient estimates. Asterisks represent significant results (*P < 0.05, **P < 0.01, ***P < 0.001). Left panel: Model output with offspring longevity as the response variable and offspring weight at weaning, quadratic maternal age, maternal weight, and date of first emergence as fixed effects (standardized; *n* = 518 individuals). Right panel: Model output with offspring lifetime reproductive success (number of yearlings reared) as the response variable and offspring weight at weaning, maternal age, maternal weight, and date of first emergence as fixed effects (standardized; *n* = 115 individuals).

**Figure 2:**
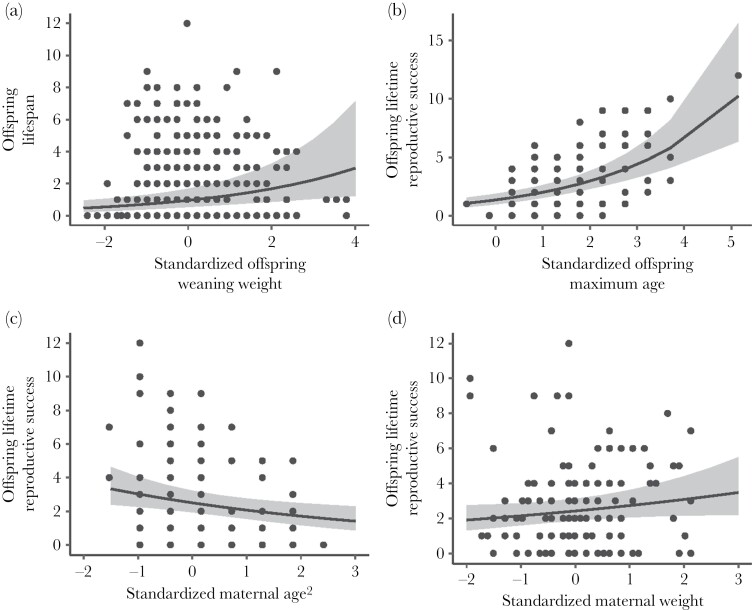
Female Columbian ground squirrel longevity (years; *n* = 518 individuals) (maximum age in years) increased with weaning weight (a). Female lifetime reproductive success (number of yearlings reared; *n* = 115 individuals) increased with offspring age (b), decreased with quadratic maternal age (c), and tended to increase with maternal weight (d). All response variables were mean-centered and standardized and are unitless, with 0 representing the mean value (a) mean offspring weaning weight was 105.56 ± 0.89 g, (b) mean offspring maximum age was 1.28 (range 0–12) years, (c) mean maternal age was 3.79 (range 2–9) years, and (d) mean maternal weight was 420.58 g (range 300–590). We plot the model-generated line of best fit with 95% confidence interval shading.

The weight of an individual at weaning had a significant effect on its longevity, with heavier individuals at weaning tending to live longer (Figures 1 and 2a; Table 1). Likewise, a female’s longevity had a significant positive effect on her LRS (Figures 1 and 2b; Table 1).

**Table 1:** Sources of variation in longevity (*n* = 518 individuals), and lifetime reproductive success (LRS; *n* = 115 individuals) for female Columbian ground squirrels. We provide point estimates from univariate mixed effects models (negative binomial with log link) for mean centered and standardized fixed effects (β) with 95 % confidence intervals (CI) and random effects. Bold values indicate statistical significance and Italics near-significance (<0.07).

	Offspring longevity	Offspring LRS
Fixed effects^a^	β (95 % CI)	β (95 % CI)
Weight at weaning	**0.28 (0.12, 0.45)**	−0.03 (−0.17, 0.12)
Maternal age^2	0.08 (−0.07, 0.24)	−**0.19 (**−**0.33,** −**0.05)**
Maternal weight	0.01 (−0.16, 0.18)	*0.12 (*−*0.01, 0.25)*
Offspring emergence date	−0.04 (−0.25, 0.18)	0.05 (−0.15, 0.24)
Offspring longevity	NA	**0.37 (0.19, 0.73)**
Random effects (σ2 ± SE)		
Maternal identity	<0.01 ± <0.01	<0.01 ± <0.01
Cohort identity/year of birth	1.08 ± 1.04	0.14 ± 0.37

^a^Mean standardized numerical variables within year.

The weight of an individual’s mother had a marginal effect on the LRS of that individual, with those born to heavier mothers having more offspring surviving to yearling age over the course of their lives (Figures 1 and 2d, Table 1).

The quadratic age of an individual’s mother at the time of birth also had a significant effect on the individual’s LRS, with individuals born to younger mothers having more yearling offspring over the course of their lives (Figures 1 and 2c; Table 1).

## DISCUSSION

Offspring weight at emergence from the natal burrow, maternal age and weight at emergence from hibernation, and longevity had significant effects on individual fitness. Individual females who were weaned heavier lived significantly longer lives than individuals weaned lighter. A potential cause of reduced longevity in smaller offspring is likely related to energy storage and expenditure. Smaller-weaned individuals may simply be too small to survive the first hibernation if they do not have sufficient fat reserves, as the pre-winter weight has been shown to influence winter survival in Columbian ground squirrels (Murie and Boag 1984). Smaller individuals may attempt to undergo a phase of accelerated growth, or “catch-up growth,” to reach a sufficient adult size (Metcalfe and Monaghan 2001). Smaller-weaned juvenile ground squirrels could potentially compensate for their weight by extending the active season to reach a condition that allows them to survive hibernation. However, this would likely lead to increased predation risk and decreased forage quality during the extended time spent above ground.

Overall, the effect of weight at weaning on longevity is supported by findings from another study on Columbian ground squirrels, showing that birth and weaning weights are important factors determining survival to yearling age (Neuhaus 2000b). Similarly, a study on yellow ground squirrels (*Spermophilus fulvus*) showed that weaning weight was the main determinant of survival to reproductive age (Vasilieva and Tchabovsky 2020). In humans, lower birth weights were also associated with higher rates of mortality (Risnes et al. 2011), linked to diabetes, coronary heart disease, or hypertension (Phillips et al. 1994; Law and Shiell 1996; Eriksson 2001; Rangel et al. 2014; Horikoshi et al. 2016).

Maternal weight did not significantly affect offspring longevity, which was surprising. One potential reason for this result may be the observation that female Columbian ground squirrels seem to delay mating and reproduce later in the season if they are of lower initial body condition (Neuhaus 2000b). Maternal weights were taken at first emergence not exactly at time of reproduction. If relatively light emerging females gain some extra weight before mating, some of the emergence weight difference would be compensated by later reproduction, leading to offspring with similar longevity to heavier, earlier reproducing females. Alternatively, lighter females may also compensate by having fewer and, therefore, on average, heavier offspring. Work in closely related Richardsons ground squirrels (*Urocitellus richardsonii*) found that females breeding relatively late in the season compensated by producing reduced litter sizes and increased individual offspring mass instead (Dobson and Michener 1995).

Future investigation into this relationship should investigate the relationship between emergence date, emergence weight, and the time between emergence and mating, or perhaps use a different metric for maternal condition, such as maternal weight gain before and during pregnancy.

According to the “Lansing Effect,” older mothers should produce offspring with reduced longevity (Lansing 1947). Contrary to our prediction, maternal age did not significantly affect offspring longevity. As will be discussed further down, offspring born to older mothers experience reduced LRS and yet appear to suffer no consequence to longevity. Columbian ground squirrels have shown reduced postnatal investment and abandonment of young that would cause an effective skipping of the breeding seasons (Neuhaus 2000b). However, future research should quantify the occurrence and frequency of litter abandonment in individuals born to older or other less favorable mothers.

Unexpectedly, weight at weaning did not significantly influence the lifetime LRS of offspring in our study. This is counter to studies that illustrate that reduced birthweights result in reduced productivity (reproductive success), for example, in humans or pigs (Lummaa 2003; Magnabosco et al. 2016). This discrepancy between our results and those of others may be a result of differences in birth weight metrics. Because we did not have access to birth weights, we used weaning weight as a proxy for birth weight, which opens the potential for homogenization of weights if individuals exhibit “catch-up growth” during lactation (Metcalfe and Monaghan 2001; Skibiel et al. 2009). We speculate that lower birth weights could be compensated by smaller litter size, leading to increased growth per offspring during lactation. Alternatively, prolonged post-emergence lactation (prolonged weaning) could also be used as compensation for small weaning weight. As females do not suckle their young above ground, we do not have any information on weaning dates. However, increased weaning weight may have indirectly affected LRS, as LRS was significantly impacted by longevity, which in turn was significantly influenced by weaning weight, a result already described in this species (Neuhaus et al. 2004). This result is congruent with other research, which shows that longevity and LRS are highly correlated (Vasilieva and Tchabovsky 2020).

The relative timing of an individual’s birth did not appear to significantly impact either longevity or LRS. However, this result is partially supported by another study on Columbian ground squirrels, which showed that the timing of reproduction and birth did not consistently affect the mass of offspring every year, effectively having little effect on the life history of the offspring (Dobson et al. 1999).

The weight and age (quadratic) of an individual’s mother appeared to have a significant effect on LRS, with heavier, younger mothers appearing to produce offspring that display higher LRS themselves. As expected, there was a strong positive correlation between longevity and LRS. This is supported by other studies in Columbian ground squirrels, which indicate that maternal body condition is an important factor in reproductive success, with individuals born to larger parents having larger litters, and therefore more offspring (Dobson et al. 1999; Skibiel et al. 2009). A study in Richardson ground squirrels found that heavier mothers tended to have larger litters with larger individual offspring (Dobson and Michener 1995). In European red squirrels (*Sciurus vulgaris*) individuals that were heavier at the age of 18 months had higher LRS (Wauters and Dhondt 1989).

Maternal age was found to significantly affect the LRS of their offspring, with older mothers producing offspring with reduced LRS. Studies in European rabbits (*Oryctolagus cuniculus*) have found somewhat comparable results, with females of a peak age (2-3 years old in rabbits) producing offspring with the highest LRS (Rödel et al. 2009), rather than a linear trend as found in our study. Our results are also consistent with studies in humans, where older mothers tend to have less fit offspring, with reduced LRS (Gillespie et al. 2013). In North American red squirrels (*Tamiasciurus hudsonicus*), senescent mothers have been shown to produce offspring of lower quality, which in turn have been linked to reduced offspring fitness (Descamps et al. 2008). According to life history theory (Stearns 1989, 1992; Monaghan et al. 2020), low-quality offspring may require more investment into factors other than reproduction, thus reducing lifetime capacity for reproduction. Senescence has been reported in populations of Columbian ground squirrels and linked to lower reproductive output (Broussard et al. 2003). Of note, the same study found that yearling females also had reduced output and reduced investment into offspring. This was not seen in our study, maybe due to a low number of yearling mothers in our population. Even so, the linear decrease in LRS with increasing maternal age is not what we expected. We suggest that the previously found strong positive effects of maternal presence on primiparous and young females’ reproductive success (King and Murie 1985; Neuhaus et al. 2004; Viblanc et al. 2016) could well be the main reason we did not observe a negative effect of young mothers on offspring LRS. Having a mother present is much more likely when the age gap between a daughter and mother is small. If this occurs, investment into female offspring at some cost to own success would be an indirect way of increasing reproductive success through inclusive fitness.

In conclusion, we found that heavier-weaned individuals experienced increased longevity but did not have a higher LRS. We also found that individuals born to heavier mothers tended to have a higher LRS but did not appear to have an increased longevity. We also found that individuals born to older mothers suffered reduced LRS but no reduction or gain in longevity. Finally, there was a strong positive correlation between longevity and LRS. Our study results add to the wealth of knowledge on life-history evolution and trade-offs in Columbian ground squirrels and expand our understanding of the importance of studying how early life conditions affect lifetime fitness in mammals. The assessment of additional or different early life conditions, such as litter size, maternal nutrition, or even climatic conditions, are valuable topics to investigate in the future. Additionally, we believe that further investigation into the mechanisms behind these trends will greatly benefit our understanding of maternal and early life history effects on individuals.

## Data Availability

Results shown in this paper can be reproduced using the excel dataset and R code provided by Tanner et al. (2024).
